# What works in global health partnerships? Reflections on a collaboration between researchers from Vietnam and Northern Ireland

**DOI:** 10.1136/bmjgh-2021-005535

**Published:** 2021-04-07

**Authors:** Chris Jenkins, Ho Thi Hien, Bui Linh Chi, Olinda Santin

**Affiliations:** 1 Centre for Public Health, Queen's University Belfast, Belfast, UK; 2 Faculty of Medicine, Hanoi University of Public Health, Hanoi, Viet Nam; 3 Queen's University Belfast School of Nursing and Midwifery, Belfast, UK

**Keywords:** health services research, health systems, other study design, descriptive study, cancer

**Summary**

- There is a growing body of literature on global health research partnerships but a lack of pragmatic and practical literature to help support researchers working in this field.
- This short commentary highlights key themes related to communication, trust and working with equality relevant for both individuals and institutions involved in collaborative global health research.
- Institutional and practical realities, along with power and financial inequalities, create challenges for the development of good and equitable relationships. These realities have to be actively addressed within research teams and between institutions.
- Good communication builds good relationships and all researchers should appreciate the importance of reflection and reflexivity within their collaborations.

## Introduction

There is a growing body of literature within global health that highlights the importance of thinking actively about how research partnerships should function. Our field is slowly coming to terms with inherent power inequalities that continue to characterise many global health research partnerships.^1–6^ It is encouraging to see the growth and breadth of articles being written on this crucially important subject.^1 5 7–9^ We felt, however, that the literature is often theoretically focused, and that a short piece discussing practical and real-world examples of factors contributing to partnership development may be beneficial for global health researchers.

This is, therefore, a short practical example of research in practice. It is not intended to be reflective of all the diverse challenges and themes within this area. It concerns our experiences of working together on cancer-related research in Vietnam.

Collaboration between the Hanoi University of Public Health and Queen’s University Belfast formally began in 2016, so this research partnership remains young and in its formative stages. These collaborations focused on first understanding challenges in breast cancer service delivery and the experiences of women with breast cancer in Vietnam. Our team came together in 2018 to build on these initial collaborations and to develop online supportive tools for people caring for someone with a cancer diagnosis.

This short piece brings together five key reflections generated by our small team of researchers on what contributes to good partnership development for the purposes of conducting high-quality global health research. The aspects of our partnership that have been effective mimic much of the literature that highlights the importance of good communication, the creation of equal responsibilities and the development of trust.^10 11^ But how were these things achieved? Such concepts are easy to write—and easy to aspire to—but often take a long time to develop and can hinge on key moments and events.

## Reflection 1: good communication needs good relationships

Good communication and good relationships go hand-in-hand. Our relationship was developed in airports, tired after cramming too much data collection into too short a time; in that strange fugue state of exhaustion combined with too much caffeine (for some of us). It was in moments waiting for delayed planes when we shared all the most vulgar and obscene of our native languages, comparing various forms of insults and the metaphors surrounding them. Our relationships were developed in these unusual, fun, in-between spaces, not in formal meetings or in ice-breaking getting-to-know-you sessions (fun as these were).

We met each other’s families and played football in the corridors of high-rise apartment blocks. We spent time visiting each other’s homes and developing an understanding of each other’s lives. We introduced each other to the biting wind and cold of the north Atlantic coastline. We sweated in Vietnamese heat and froze in an Irish winter. We balanced unconvincingly on the back of motorbikes. And we developed our relationships while working in hotel rooms overlooking the Mekong River, painstakingly line-by-line editing our collaborative papers, checking our data and deepening our understanding of it.

The development a good relationship was important in the infancy of our project as, like many projects, we had not yet been awarded a large grant to support our work. Initial paper writing was done with a view to longer term goals and to demonstrate solid outputs from seed funding. These activities were, however, unfunded in and of themselves. Good relationships, communication and the establishment of trust between our team allowed us to effectively navigate this potentially derailing time.

We worked through COVID-19 and our relationship was relocated to Zoom. The grounding we had developed allowed this to happen relatively seamlessly. We invested our time in weekly virtual meetings. We met regularly, even though Zoom can be exhausting. We kept each other accountable and tracked the progress we were making. We codeveloped a forward plan of action and sought funding to enable it. Our good relationships, and the trust inherent within them, gave us a base of transparency, honesty and comfort to communicate openly throughout all these different phases of our projects and to meet a challenge like COVID-19 head-on.

The literature on communication and trust underlines the importance of such concepts in partnership building. But you cannot develop these concepts by wishing them into place. You develop them through friendships, fun and inappropriate language sharing. You do it by investing time into them, but without ever explicitly viewing it as an investment of time. We met each other’s dogs. We met each other’s cats.

## Reflection 2: we all need to eat

We ate a lot. Food, as many anthropologists have noted, is central to how many people interact. We discovered different foods and enjoyed the differences in each other’s cuisines. Food opens up conversations and provides an insight into different cultures and histories. It can provide fun and laughter in new experiences. We delved into huge multicourse Vietnamese lunches. We shared recipes. Our Northern Irish members felt ashamed when trying to convince research partners that a pint of Guinness and a bag of crisps is considered an acceptable meal in Belfast. We argued over shrimp paste and fish sauce. We planned culinary back-up careers and opening Vietnamese restaurants in Ireland. We guided each other and ensured that we did not embarrass each other with horrendous chopstick etiquette.

Eating is a time when you can take the foot off the peddle, relax a little and ask each other questions beyond work. Eating is not peripheral to good relationship development but is at its core. It, therefore, should be viewed as a key part of research development that time and energy are invested into.

Beyond relationship development, eating is also a time to unpack wider themes that may relate to your research. It provides space to talk about social and cultural norms, history, current affairs, the media, language and all the other many fields that often impact the data we collect during the day. We learn as we eat.

## Reflection 3: work with equality

Again, this one should be obvious. But, similar to achieving good communication, working with equality can be easy to say and much harder to do. This is especially the case given that we all live and work in societies and contexts that are shaped by inequalities and remain unequal to this day. Good relationships help encourage equality but are not enough in themselves.

We have to talk about equitable outcomes, respect and supportive environments. We cannot just assume they will happen. What does each partner need and want from the project? How are outputs like authorship divided? What support is needed to ensure such outputs are fairly distributed? What are our expectations from each other?

Financial inequality is an obvious reality and challenge to overcome in creating equal relationships. We did our best to ensure that money from grants was divided between the partners and discussed in depth how to appropriately resource the work we were planning on undertaking. We endeavoured to ensure a fair split in authorship. We explicitly booked time and space to write together and support members of the team with less experience of leading authorship. We identified different training and educational opportunities, including opening up funding for PhD study. We shared speaking opportunities at conferences. We ensured that travel opportunities were reciprocal.

At times we have not held to high enough standards regarding some of our aspirations. Our weighting of international/Vietnamese lead authorship is not sufficiently balanced (it should of course, not be balanced but weighted in favour of authors from Vietnam^12^). Timeline pressures, at times, meant we took the quicker option of having a native-English speaker write papers. At times, Vietnamese partners had too much other work to take the time to lead authorship, and we did not sufficiently or adequately open up that time, space and support for them to do that.

This partnership, like all partnerships, still has room for growth and improvement. Hopefully, we can view that as something exciting to work towards.

## Reflection 4: institutions are important

Within our wider group, we also had incredible support from more senior researchers within each university, who may not have shared in all of the moments described in this paper but who created an atmosphere in which we all felt supported. They took us out for dinner, showed incredible generosity and also invited us to meet their families and share stories. Their support, leadership, vision and direction were qualities consistently mentioned by our research team as underpinning our partnership.

Institutional and funding barriers, however, can also make having genuine, equitable, partnerships difficult. The various reporting requirements of our different legal, financial and administrative departments, while often important for providing good structural grounding and accountability for projects, often reinforce inequalities and undermine trust building. By channelling funding through the international partner, this can create a feeling of ‘financial nannying’ and paternalism that is not helpful, equitable or efficient.

Processes may be straightforward in one institution but may be a minefield in another. Not understanding these differences, or making assumptions about how institutions can and/or should function, can lead to unhelpful and unfair frustration. Learn about each other’s institutions. Yes, most of us are researchers, first and foremost. But we also have to be managers and accountants. We have to understand legal contracts and human resources. Most importantly, we have to be able to translate between all these groups and across institutions. It's essential to build in time for these stressful and time-consuming aspects of project management.

## Reflection 5: reflect!

Take time to think about your partnerships, how they currently function and what you want them to look like. Talk to each other. Listen to each other. Build in the time and space to do this. Keep a research diary. Interrogate your own project. Reflective and reflexive projects are good projects and are essential to also producing good research.^13–16^

## Conclusions

For all these reflections and the successes that we believe we have had through this collaborative work, none of this is to say that there are not problems in our partnership. Partnerships are complex. We do some things well, and others poorly. We reflect and accept that in hindsight we may have done some things differently.

We hope that this short commentary of practical real-world experiences of one partnership helps provide some food-for-thought and pointers for researchers either embarking on new collaborative projects or seeking to take a step back and reflect on their own practice.

## Data Availability

No data are available.
